# Correlative studies of the Breast Cancer Index (HOXB13/IL17BR) and ER, PR, AR, AR/ER ratio and Ki67 for prediction of extended endocrine therapy benefit: a Trans-aTTom study

**DOI:** 10.1186/s13058-022-01589-x

**Published:** 2022-12-16

**Authors:** Dennis C. Sgroi, Kai Treuner, Yi Zhang, Tammy Piper, Ranelle Salunga, Ikhlaaq Ahmed, Lucy Doos, Sarah Thornber, Karen J. Taylor, Elena Brachtel, Sarah Pirrie, Catherine A. Schnabel, Daniel Rea, John M. S. Bartlett

**Affiliations:** 1 Molecular Pathology Research Unit, Department of Pathology, Harvard Medical School, Massachusetts General Hospital East, 149 13th Street, Charlestown, MA 02129 USA; 2 Massachusetts General Hospital Center for Cancer Research, Harvard Medical School, Boston, MA USA; 3 Biotheranostics, A Hologic Company, San Diego, CA USA; 4 University of Edinburgh, Edinburgh, UK; 5 Cancer Research UK Clinical Trials Unit, University of Birmingham, Birmingham, UK; 6 Ontario Institute for Cancer Research, Ontario, Toronto, ON Canada; 7 University of Toronto, Toronto, ON Canada

**Keywords:** Breast Cancer Index, BCI (H/I), Breast cancer, Predictive biomarker, Extended endocrine therapy

## Abstract

**Background:**

Multiple clinical trials demonstrate consistent but modest benefit of adjuvant extended endocrine therapy (EET) in HR + breast cancer patients. Predictive biomarkers to identify patients that benefit from EET are critical to balance modest reductions in risk against potential side effects of EET. This study compares the performance of the Breast Cancer Index, BCI (*HOXB13*/*IL17BR*, H/I), with expression of estrogen (ER), progesterone (PR), and androgen receptors (AR), and Ki67, for prediction of EET benefit.

**Methods:**

Node-positive (N+) patients from the Trans-aTTom study with available tissue specimen and BCI results (*N* = 789) were included. Expression of ER, PR, AR, and Ki67 was assessed by quantitative immunohistochemistry. BCI (H/I) gene expression analysis was conducted by quantitative RT-PCR. Statistical significance of the treatment by biomarker interaction was evaluated by likelihood ratio tests based on multivariate Cox proportional models, adjusting for age, tumor size, grade, and HER2 status. Pearson’s correlation coefficients were calculated to evaluate correlations between BCI (H/I) versus ER, PR, AR, Ki67 and AR/ER ratio.

**Results:**

EET benefit, measured by the difference in risk of recurrence between patients treated with tamoxifen for 10 versus 5 years, is significantly associated with increasing values of BCI (H/I) (interaction *P* = 0.01). In contrast, expression of ER (*P* = 0.83), PR (*P* = 0.66), AR (*P* = 0.78), Ki67 (*P* = 0.87) and AR/ER ratio (*P* = 0.84) exhibited no significant relationship with EET benefit. BCI (H/I) showed a very weak negative correlation with ER (*r* = − 0.18), PR (*r* = − 0.25), and AR (*r* = − 0.14) expression, but no correlation with either Ki67 (*r* = 0.04) or AR/ER ratio (*r* = 0.02).

**Conclusion:**

These findings are consistent with the growing body of evidence that BCI (H/I) is significantly predictive of response to EET and outcome. Results from this direct comparison demonstrate that expression of ER, PR, AR, Ki67 or AR/ER ratio are not predictive of benefit from EET. BCI (H/I) is the only clinically validated biomarker that predicts EET benefit.

**Supplementary Information:**

The online version contains supplementary material available at 10.1186/s13058-022-01589-x.

## Background

Extending the duration of endocrine therapy from 5 to 10 years has been demonstrated to provide consistent but modest benefits to breast cancer patients in the range of 1% to 5% in patients with hormone receptor-positive (HR+) early stage breast cancer [1, 2]. HR + patients treated with extended endocrine therapy exhibited a significant reduction in the risk of late recurrence [3–5]. However, gains in risk reduction must be balanced against serious adverse events, such as bone and cardiovascular toxicities, endometrial cancer, and other adverse effects that are associated with the use of anti-estrogen therapies [3]. About 30–50% of HR + tumors fail to respond to endocrine therapy, while over 50% of recurrences occur more than 5 years after diagnosis [6–8]. Predictive biomarkers are essential to identify the subset of patients who are likely to benefit from extended endocrine therapy.

Estrogen receptors (ER), progesterone receptors (PR), androgen receptors (AR), and Ki67 are biomarkers with demonstrated clinical value in breast cancer. ER and PR expression, measured quantitatively, are canonical biomarkers for classifying tumors as potentially endocrine sensitive and therefore eligible for endocrine therapy [9]. However, about 30–50% of ER-positive patients do not respond to endocrine therapy [8, 10, 11]. Further, translational studies in randomized cohorts from the Breast International Group 1–98 (BIG 1–98), Arimidex, Tamoxifen, Alone or in Combination (ATAC), and Tamoxifen Exemestane Adjuvant Multinational (TEAM) clinical trials have demonstrated that quantitative ER or PR expression are not predictive biomarkers for endocrine response [12–14].

AR is reported as being positive in 60–90% of primary breast cancers [15]. Whether AR levels have predictive value for endocrine therapy remains controversial [16–18]. Additionally, AR and ER expression values, combined as a ratio (AR/ER), have been suggested to predict failure of endocrine therapy [19, 20]. Ki67 is a nuclear protein that is expressed in all proliferative phases of cell division (late G1, S, G2, and M). It has been shown to be prognostic in early stage, ER-positive/HER2-negative breast cancer [21], but is only moderately predictive of distant recurrence [7, 22].

The Breast Cancer Index (BCI) is a gene expression-based signature comprising two functional biomarker panels [23, 24]. The Molecular Grade Index (MGI) is composed of five genes that measure tumor proliferation. BCI (H/I) is a ratio of the *HOXB13* and *IL17BR* genes and measures estrogen signaling. Integration of MGI and BCI (H/I) provides a single prognostic score that quantifies the risk of both late (5–10 years) and overall (0–10 years) distant recurrence. BCI (H/I) has been validated to predict benefit from extended endocrine therapy (EET) across multiple adjuvant endocrine treatment backgrounds in several prospective-retrospective studies [24–26].

The objective of this correlative biomarker study was to directly compare the predictive value of BCI (H/I) with ER, PR, AR, and Ki67 protein expression and the AR/ER ratio in a large cohort of patients treated in the Adjuvant Tamoxifen—To Offer More (aTTom) clinical trial (or the Trans-aTTom cohort) with respect to extended endocrine therapy.

## Methods

### Aims

This study compares the performance of BCI (H/I), with expression of ER, PR, AR, and Ki67, for prediction of benefit from EET in a cohort of patients from the translational aTTom study. The translational aTTom study, Trans-aTTom, is a multi-institutional, prospective-retrospective study with the objective of validating BCI (H/I) as a predictive biomarker of EET benefit in patients treated in the aTTom trial [24]. The University of Birmingham Cancer Research UK Clinical Trial Unit (CRCTU) was the sponsoring institution and secured ethical and regulatory approvals from the UK Research Ethics Committee (REC, reference 16/EM/0142), Health Research Authority (HRA), Confidentiality Advisory Group (CAG) and from the PBPP in Scotland, and also carried out final biomarker data integration with the aTTom clinical database.

### Patients and tumor samples

**Fig. 1:**
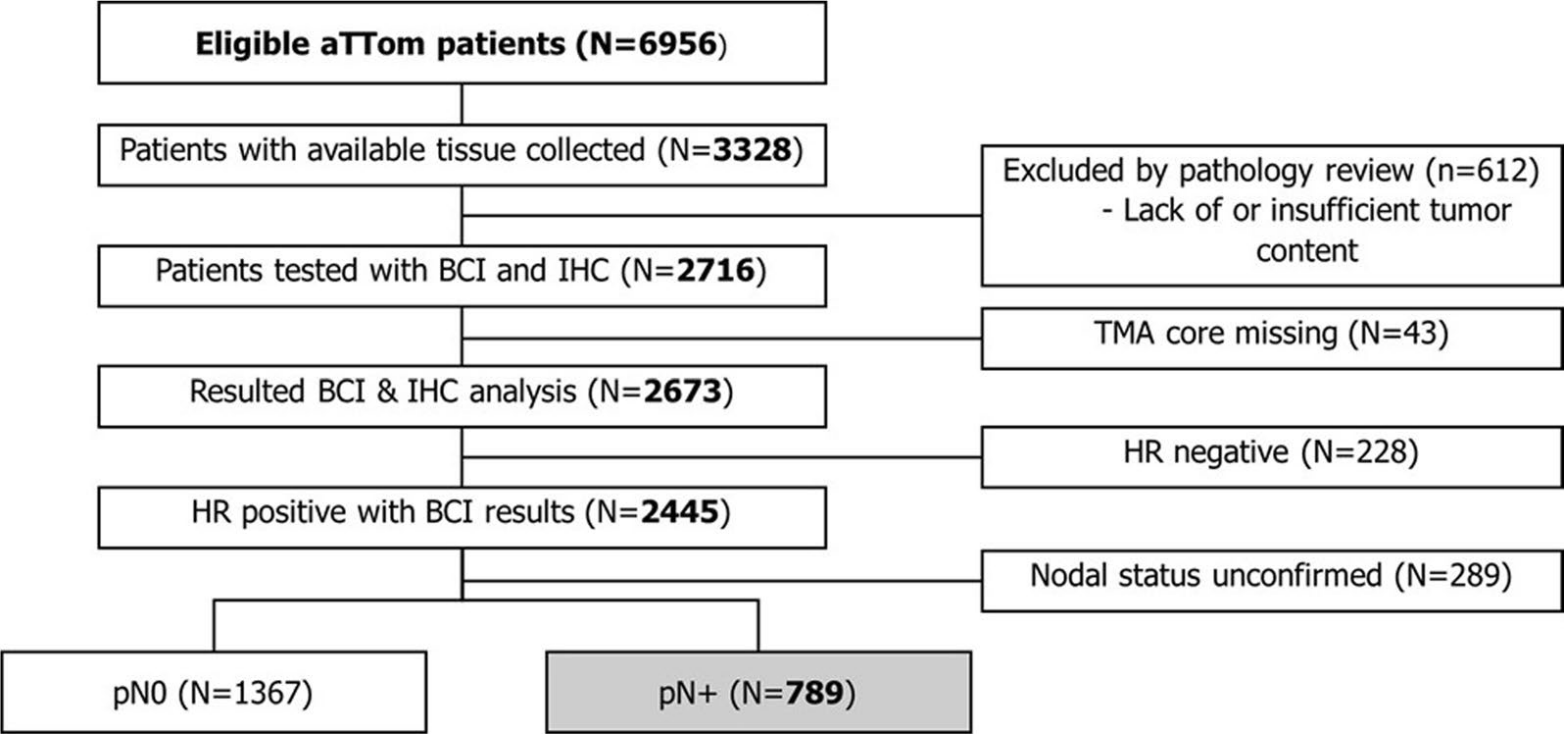
Patient case flow for the Trans-aTTom study. The diagram shows tumor block collection, specimen processing, IHC and molecular testing, leading to a final analyzable cohort of 789*N* + patients

The parent aTTom trial is a prospective, phase III trial that included 6956 breast cancer patients who remained disease free after having completed at least 4 years of adjuvant tamoxifen therapy and were randomized to either continue or stop tamoxifen treatment of an additional 5 years [27]. All patients previously randomized in the aTTom trial with available formalin-fixed paraffin-embedded (FFPE) primary resection tumor blocks were included in the study. Exclusion criteria included absence of invasive tumor as evaluated by histopathology review, insufficient tumor on tissue microarray (TMA) for immunohistochemical (IHC) analysis, and insufficient RNA for BCI analysis (Fig. 1). Centralized collection and sample processing, construction of TMAs, and tissue sectioning was carried out by the University of Edinburgh Cancer Research Center (ECRC) as described previously [24].

Following the initial disclosure of the Trans-aTTom results reporting significant prediction of extended endocrine benefit by BCI (H/I) in node-positive (*N*+) patients [24], case collection continued in a pre-specified and blinded manner due to insufficient power of < 50% estimated for both the overall cohort and the node-negative (*N*0) subset. After the completion of block collection (*N* = 3328), however, insufficient power prevented an analysis in the overall cohort and N0 subset. Therefore, the current analysis is based on 789 *N*+ patients from the final Trans-aTTom cohort.

### Immunohistochemical analysis

Two 1-mm cores were obtained from each tumor block and duplicate TMAs were constructed. IHC staining of the TMAs was performed in a CLIA-certified laboratory at Massachusetts General Hospital using the Leica Bond III Autostainer (Leica Microsystems, Inc., Buffalo Grove, IL, USA). ER and PR were detected using Clone 6F11 (Leica Biosystems, RRID:AB_2827388) and Clone 16 (Leica Biosystems, RRID:AB_10554439), respectively. AR and Ki67 were detected using Clone AR441 (GeneTex, RRID:AB_367520) and Clone K2 (Leica Biosystems, RRID:AB_2341199), respectively. TMAs were reviewed centrally and scored visually by two board-certified breast pathologists (DCS, EFB) who were blinded to all clinicopathological, BCI and outcome data. ER and PR were independently assessed by two pathologists (DCS, EFB), while AR and Ki67 were assessed by one board-certified pathologist (DCS). ER and PR expression were scored in accordance with ASCO/CAP testing guidelines [9]. AR expression was scored in a manner similar to ER and PR using the same ≥ 1% cut-off for AR positivity. An Allred score that captures both the proportion and intensity of staining for ER, PR and AR was determined following the methodology described [28]. Ki67 was scored as percentage of positively staining tumor cell nuclei using the International Ki67 in Breast Cancer Working Group “global” method [29].

### BCI assay

BCI gene expression analysis by RT-PCR was carried out on formalin-fixed paraffin-embedded (FFPE) primary tumor specimens (Biotheranostics, A Hologic Company, San Diego, CA) blinded to clinical outcome as reported previously [30]. Briefly, macro-dissection was performed on FFPE sections to enrich tumor content before RNA extraction. Total RNA was reverse transcribed, and the resulting cDNA was pre-amplified by PCR using the PreAmp Master Mix Kit (Thermo Fisher Scientific, Carlsbad, CA) before TaqMan PCR analysis. Calculation of BCI (H/I) was carried out as described previously and was normalized into a range between 0 and 10 [23, 26].

### Statistical analysis

Analyses were pre-specified in the statistical analysis plan. The primary endpoint was recurrence-free interval (RFI), defined as the time from randomization to first local, regional, or distant recurrence. The statistical power for the *N* + patients was 94% to detect an interaction of extended adjuvant tamoxifen by BCI (H/I) status at 0.05 significance level for the primary endpoint (RFI). The secondary endpoints were disease-free interval (DFI), defined as the time from randomization to first local, regional, distant recurrence, or new breast primary, and disease-free survival (DFS), defined as time from randomization to first local, regional, distant recurrence, new breast primary, or breast cancer death. Distribution of each biomarker was graphically presented by histograms. Pearson’s correlation coefficients and scatterplots were calculated to evaluate correlations between BCI (H/I) versus ER, PR, AR, Ki67 and AR/ER ratio. Cox proportional models were used to evaluate the relationship of the risk of recurrence as a function of continuous biomarker values in each treatment arm. The statistical significance of the treatment by biomarker interaction was assessed by likelihood ratio tests based on multivariate Cox proportional models, adjusting for age, tumor size, grade and HER2 status.

## Results

### Patient demographics and characteristics

Primary tumor specimens from 3328 aTTom patients were collected retrospectively (Fig. 1) [31]. Of the 2445 HR + patients with BCI results, 789 (32%) had node-positive (*N*+) disease. In the *N*+ subset, 87% (688/789) were ≥ 50 years old and 86% (679/789) were postmenopausal (Table 1). A total of 89% (698/789) of these patients had T1 or T2 tumors. Among *N*+ patients, 98% (771/789) had ER-positive disease and 91% (717/789) had PR-positive disease. HER2 positivity was confirmed in 9% (72/789) of patients. There were 43 and 32 local recurrences, and 113 and 94 distant recurrences in the 5-year and 10-year tamoxifen arms, respectively. No statistically significant differences were observed in clinical variables for *N*+ patients between the parent aTTom trial and the Trans-aTTom study.

**Table 1:** Clinicopathological characteristics for HR + *N* + patients in the Trans-aTTom cohort

	Trans-aTTom HR + pN + (*N* = 789)
*Age*	
< 50	101 (13%)
50–59	272 (34%)
60–69	218 (28%)
≥ 70	198 (25%)
*Menopause status*	
Pre	25 (3%)
Post	679 (86%)
Peri	28 (4%)
Not known	57 (7%)
*Tumor size*	
*T*1	362 (46%)
*T*2	336 (43%)
*T*3	30 (4%)
Not known	61 (8%)
*Histological grade*	
Well differentiated	118 (15%)
Moderately differentiated	369 (47%)
Poorly differentiated	161 (20%)
Not known	141 (18%)
*ER status*	
Negative	17 (2%)
Positive	771 (98%)
Not known	1 (0%)
*PR status*	
Negative	69 (9%)
Positive	717 (91%)
Not known	3 (0%)
*HER2 status*	
Negative	711 (90%)
Positive	72 (9%)
Not known	6 (1%)

### Distribution of biomarker expression levels

**Fig. 2:**
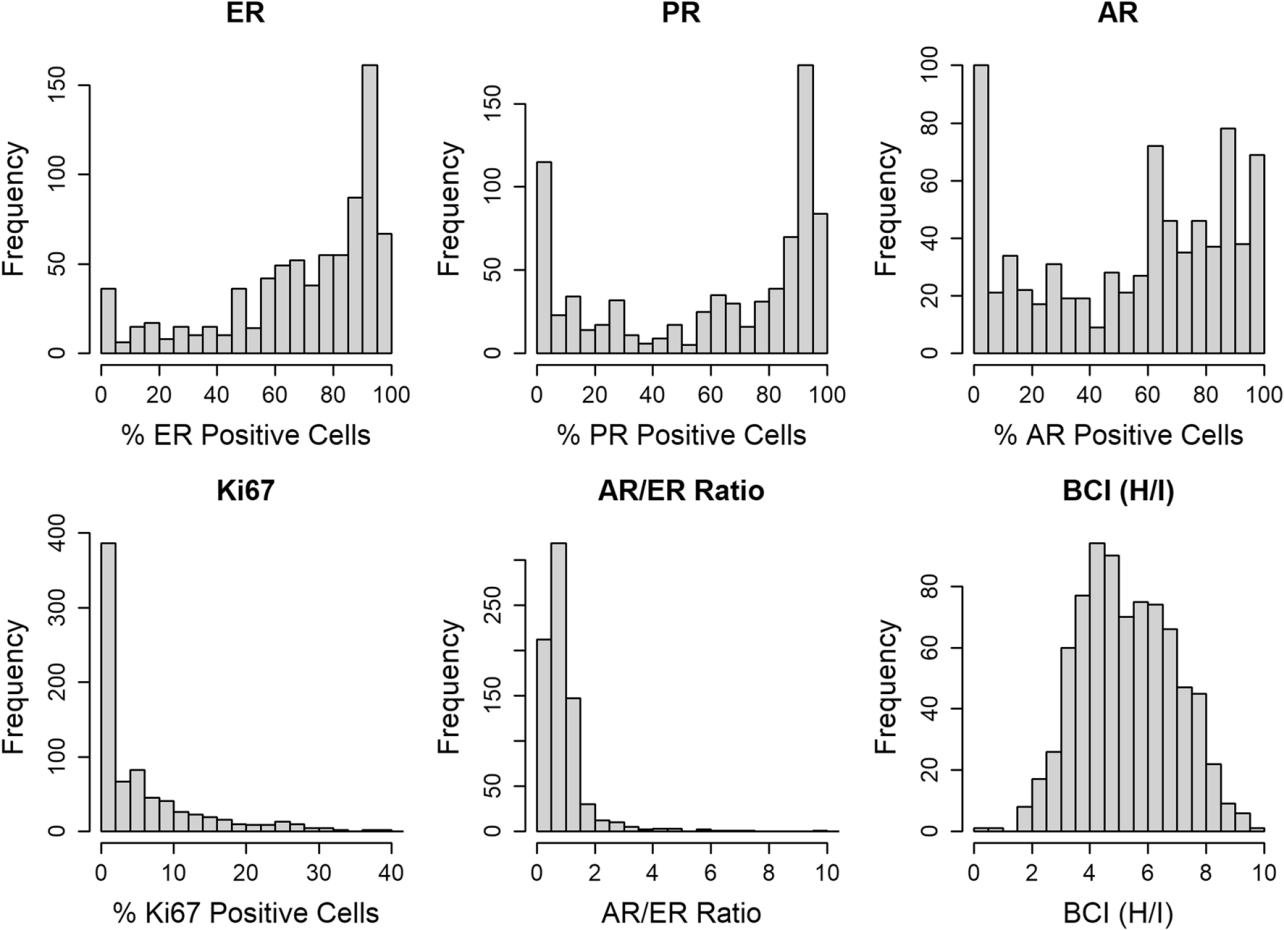
Distribution of biomarker expression in breast cancer tumors. Proportion of cells depicted as percent (%) immunohistochemical positivity for estrogen receptor (ER), progesterone receptor (PR), androgen receptor (AR), Ki67 AR/ER ratio and BCI (H/I) values

Each biomarker assessed in this study exhibited a distinct distribution of expression levels (Fig. 2). For ER, the distribution was unimodal with a prominent peak of tumors displaying ≥ 80% ER-positive cells. PR exhibited a bimodal distribution, with a peak at ≥ 80% of tumor cells staining positive for PR, and another peak at < 10%. Similarly, the distribution of AR was bimodal with a broad peak at > 60% of tumor cells staining positive for AR, and another peak at < 10%. In contrast, the distribution of Ki67 was different from the other biomarkers with a majority of tumors displaying a low percentage of Ki67-positive cells. The majority of tumors exhibited an AR/ER ratio < 1. BCI (H/I) results, normalized to a scale from 0 to 10, displayed an approximately Gaussian distribution with a median value of 5.1.

### Benefit from extended tamoxifen is correlated with increasing levels of BCI (H/I)

**Fig. 3:**
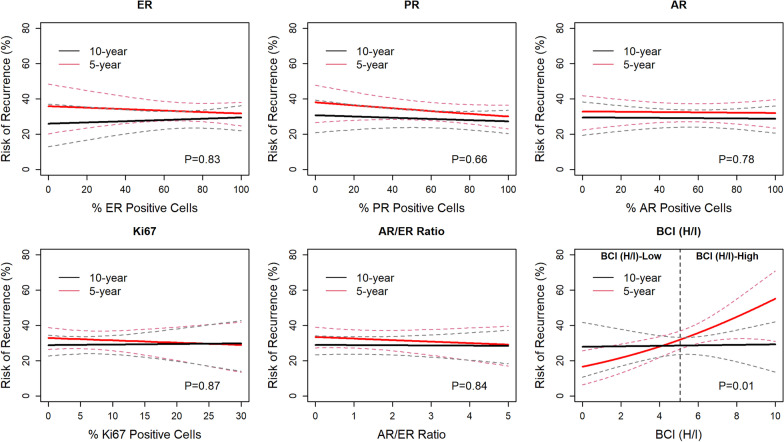
Benefit from 10 versus 5 y Tamoxifen is associated with increasing levels of BCI (H/I). Continuous risk curves based on RFI after 5- and 10 years of tamoxifen as a function of estrogen receptor (ER), progesterone receptor (PR), androgen receptor (AR), and Ki67 immunohistochemistry (IHC) expression, AR/ER IHC ratio and BCI (H/I) values

The objective of this study was to investigate potential relationships between benefit from extended endocrine benefit and each biomarker by assessing the statistical significance of treatment by biomarker interaction. The results of these analyses showed that there was no significant interaction between extended tamoxifen treatment and expression of ER (*P* = 0.83), PR (*P* = 0.66), AR (*P* = 0.78), Ki67 (*P* = 0.87), or the AR/ER ratio (*P* = 0.84) for the primary endpoint RFI (Fig. 3) or the secondary endpoints DFI (Additional file 1: Fig. 1) and DFS (Additional file 1: Fig. 2), demonstrating that these biomarkers did not predict the benefit of 10 versus 5 years of endocrine therapy. In contrast, the magnitude of extended endocrine benefit was associated with increasing levels of BCI (H/I) (Fig. 3, lower right corner). The interaction between BCI (H/I) and extended endocrine treatment was statistically significant for RFI (*P* = 0.01), DFI (*P* = 0.01), and DFS (*P* < 0.01), when corrected for age, tumor size, grade, ER, and PR status.

There was no significant interaction between the extended tamoxifen treatment and the combined proportion and intensity of ER (*P* = 0.22), PR (*P* = 0.29) and AR (*P* = 0.26) expression as determined by the Allred scoring system (Additional file 1: Fig. 3). Similarly, no significant interaction was seen between endocrine treatment and ESR1 (*P* = 0.12) or PR (*P* = 0.45) mRNA expression. The benefit analysis shown in Fig. 3 was also performed for the overall HR + cohort (*n* = 2445) and is included for comparison (Additional file 1: Fig. 4).

**Fig. 4:**
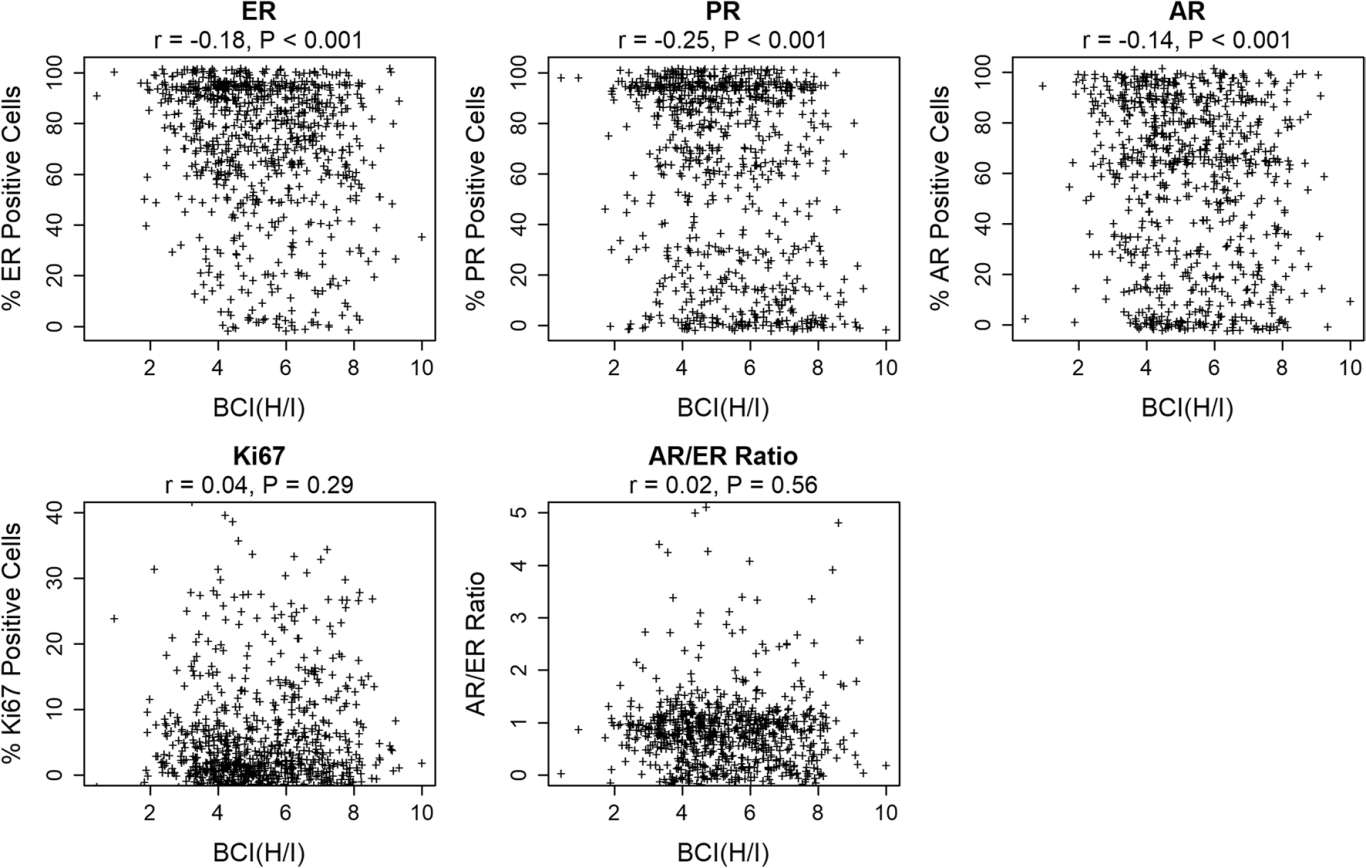
BCI (H/I) shows weak negative correlation with ER, PR and AR and No Correlation with Ki67 or AR/ER ratio. Pearson’s correlation coefficients (*r*) for estrogen receptor (ER), progesterone receptor (PR), androgen receptor (AR), AR/ER ratio and BCI (H/I)

### Correlation of BCI (H/I) with ER, PR, AR, Ki67, and AR/ER ratio

The strength and direction of possible relationships between BCI (H/I) and the other biomarkers were further assessed using Pearson correlation coefficients. BCI (H/I) exhibited weak negative correlations with the percent positive cells stained for ER (*r* = − 0.18, *P* < 0.001), PR (*r* = − 0.25, *P* < 0.001) and AR (*r* = − 0.14, *P* < 0.001) (Fig. 4). In addition, no relationship was observed between BCI (*H*/*I*) and the percent of Ki67 expressing cells (*r* = 0.04, *P* = 0.29) or the AR/ER ratio (*r* = 0.02, *P* = 0.56).

### BCI is prognostic for late distant recurrence

Of the biomarkers evaluated in this study, only BCI was significantly prognostic for late distant recurrence (interquartile HR 1.37, *P* < 0.001; see Additional file 1: Table 1). ER protein (interquartile HR 0.92, *P* = 0.165), PR protein (interquartile HR 0.89, *P* = 0.281), AR protein (interquartile HR 0.87, *P* = 0.120), Ki67 protein (interquartile HR 0.96, *P* = 0.489), the AR/ER ratio (interquartile HR 0.99, *P* = 0.484), ER mRNA (interquartile HR 1.05, *P* = 0.441), and PR mRNA (interquartile HR 1.02, *P* = 0.811) did not provide statistically significant prognostic information.

## Discussion

The findings presented here demonstrate that of the biomarkers comparatively assessed in the Trans-aTTom study, BCI (H/I) is the only biomarker predictive of extended endocrine benefit. ER, PR, AR, Ki67, and the AR/ER ratio were found to have no significant clinical value for predicting benefit from extended endocrine therapy.

The quantitative immunohistochemical findings reported here both as proportion of cells, and the combination the proportion and intensity of staining of cells (Allred score), are also consistent with results from other trials indicating that neither ER nor PR are predictive of benefit from extended endocrine therapy [12–14]. The ability of quantitative expression levels for ER and PR to predict benefit from letrozole over tamoxifen was examined in the BIG 1–98 trial [13]. Both ER and PR were found to be prognostic, but neither biomarker predicted benefit from extended endocrine therapy with respect to DFS. The ATAC trial assessed the ability of quantitative expression levels of ER and PR to predict benefit from anastrozole over tamoxifen with respect to time to recurrence; however, neither ER nor PR were found to be predictive of benefit [12]. In addition, results from the TEAM trial demonstrated that PR expression levels are not predictive of benefit from exemestane over tamoxifen with respect to DFS, and that ER is not predictive of preferential benefit from exemestane over tamoxifen [14].

The current results further highlight the unique clinical value of BCI (H/I) as a predictive biomarker of endocrine benefit. BCI (H/I) provides distinct information regarding tumor biology, as evidenced by the lack of correlations with ER, PR, AR and Ki67 (Fig. 4). BCI (H/I) interrogates estrogen signaling by evaluating the ratio of *HOXB13* and *IL17BR* gene expression, with *HOXB13* expression potentially being the primary determinant of predictive activity. Transient *HOXB13* overexpression in breast cancer cells was shown to rapidly reprogram and expand the binding pattern of ER to genomic regions that were previously inaccessible and inactive. Genomic regions newly bound by ER are enriched for genes involved in mammary gland development and differentiation. HOXB13 and ER binding sites are colocalized in about 50% of these genomic regions, suggesting a role for HOXB13 in transcriptional regulation of ER [32]. The collocation of DNA binding sites provides an opportunity for HOXB13 and ER to influence the expression of each other. Thus, BCI (H/I) provides additional functional information on estrogen signaling and response to endocrine therapy beyond that provided by expression of ER and/or PR alone.

Previous studies suggested that low ESR1 mRNA levels are associated with tamoxifen resistance in ER-positive breast cancer [33]. ESR1 mRNA levels were shown not to predict benefit from extended endocrine benefit in the MA.17 trial [26]. More recently, ESR1 mRNA levels have been evaluated as a potential predictor of response to extended endocrine benefit in the NSABP B-42 trial [34]. The results of the analysis showed no significant interaction between the response to extended letrozole therapy and ESR1 mRNA levels. The results presented here are consistent with these previous results and do not support the use of ESR1 expression as a predictive biomarker of response to extended endocrine benefit. These findings indicate that ESR1 mRNA levels alone are biologically not relevant to prediction of extended endocrine benefit, but rather point to the importance of modulating estrogen signaling by HOXB13 and potentially other cofactors, including FOXA1 and GATA-3 [35, 36]. This mechanism may allow HOXB13 to contribute to ER reprogramming in early stage breast cancer. Such functional reprogramming may then lead to differential responses to endocrine therapy.

It should be noted that even though neither protein expression nor mRNA expression of ER were significant predictors of benefit from 10-year tamoxifen therapy, their expression patterns in relation to the benefit of extended endocrine therapy appear to be distinct: there was no differential benefit across the range of ER protein expression levels; however, higher mRNA expression of ER was associated with numerically less benefit from extended endocrine therapy. The difference may reflect the low concordance between protein and mRNA expression levels often observed for many biomarkers [37].

The clinical value of AR as a biomarker in breast cancer continues to be debated. AR levels have been reported as significant predictors of response to primary adjuvant endocrine therapy, but only under certain circumstances: either in triple negative breast cancer or when only specific genotypes of AR are considered [38, 39]. On the other hand, AR alone has been reported to lack clinical utility for predicting response to primary adjuvant endocrine therapy in HR + tumors [16, 17]. AR has not previously been examined as a predictive biomarker for extended endocrine benefit.

Combining AR and ER into an AR/ER ratio may provide additional information, and previous reports suggest that an AR/ER ratio ≥ 2 is associated with increased resistance to endocrine therapy, as well as being a marker of increased cellular proliferation [19, 40]. The majority of tumors in the current study exhibited an AR/ER ratio < 2 (Fig. 3), suggesting that the majority of patients would respond to endocrine therapy and exhibit less resistance. However, there was no statistically significant relationship between extended tamoxifen benefit and the AR/ER ratio in this study (*P* = 0.83).

Due to the strong correlation between S phase and Ki67 expression levels, quantitative evaluation of Ki67 can provide a precise estimate of tumor proliferation [16, 17]. Even though there is no significant relationship between Ki67 expression levels and either hormone receptors or HER2, Ki67 has been shown to be prognostic in early-stage ER-positive/HER2-negative breast cancers [21]. As a marker of proliferation, Ki67 is strongly correlated with tumor size and nodal status but is only moderately predictive of distant recurrence [7, 22]. In the monarchE trial, Ki67 was found to be prognostic but not predictive of response to adjuvant abemaciclib [41]. Consistent with these previous results, Ki67 was observed in this study to have no value for predicting response to EET. In the current study, Ki67 levels were determined as a percentage of positively staining tumor cell nuclei using the International Ki67 in Breast Cancer Working Group “global” method [29] on TMAs, with each tumor represented by two 1-mm histospots taken from two different locations within each tumor block. Use of TMAs may have contributed to lower-than-expected Ki67 scores, in that it involves sampling less of the tumor. However, two 0.6-mm histospots have been shown to adequately represent sampling from a complete tumor section [42]. A more likely explanation is that the parent aTTom trial only enrolled patients that had remained recurrence free after at least 4 years of adjuvant endocrine therapy [24], in that less than half of recurrences occur within 5 years of diagnosis [6, 7, 43]. Furthermore, any potential systematic bias would be expected to be uniform across the entire cohort. Because the Ki67 analysis was based on continuous Ki67 measurements, any potential bias is unlikely to have affected the results in a significant manner.

The limitations of this study include applying a clinically actionable threshold for ER, PR, AR, and Ki67 expression, particularly in regard to inconsistent cutoffs used to determine Ki67 positivity [9]. ASCO/CAP guidelines define tumors to be ER-positive if ER staining is observed in at least 1% of tumor nuclei; however, data are limited regarding benefit from endocrine therapy for patients with less than 10% of tumor cells staining positive for ER. Further, quantitation of ER and PR present technical challenges that can lead to varying or inconsistent interpretation of results [16, 17].

Standardization of Ki67 measurement has been the goal of an international working group, which has produced recommendations to standardize laboratory procedures and to improve the analytical validity of Ki67 measurements [29]. Although significant methodological improvements have been made, analytical validity continues to be a concern, affecting both the clinical utility of Ki67 and the ability to compare different studies [29]. Thus, some studies suggest that Ki67 may be predictive of response to adjuvant endocrine therapy [44], whereas other studies find that Ki67 is not predictive [45–47], indicating that no consensus has been reached. In the current study, no correlation was found between Ki67 expression and response to extended endocrine therapy, indicating that Ki67 may be of limited use for clinical decision-making regarding extended endocrine benefit.

The results presented here highlight an important distinction between prognostic and distinctively predictive biomarkers in breast cancer. While the estimation of recurrence risk is an important factor in the consideration of extended endocrine therapy, prognostic factors are inadequate for determining whether patients should receive extended endocrine therapy; biomarkers predictive of response to endocrine therapy should be used as well. About 30–50% of HR + breast cancer tumors are resistant to endocrine therapy [8, 10, 11]. Endocrine resistance may contribute to recurrences, which occur at a steady rate of 1–2% per year, with at least 50% of recurrences occurring more than 5 years following diagnosis [43]. Further, endocrine resistance may also alter sensitivity to subsequent chemotherapies [48]. Resistance to therapy and distant metastases are the leading causes of death in breast cancer [8]. Thus, it is critical that truly predictive biomarkers are used to identify those patients most likely to respond to EET. BCI has demonstrated its clinical value in predicting benefit from EET and has been included as the only predictive biomarker of extended endocrine benefit in both the ASCO and NCCN clinical practice guidelines [49, 50].

## Supplementary Information

**Additional file 1. Figure 1**: DFI Benefit From 10 vs 5 y Tamoxifen is associated with increasing BCI (H/I) Levels. **Figure 2**: DFS benefit from 10 vs 5 y Tamoxifen is associated with increasing BCI (H/I) levels. **Figure 3**: Extended endocrine benefit from 10 vs 5 y Tamoxifen is not associated with Allred scores of ER, PR, or AR , or expression of ER or PR mRNA. **Figure 4**. Extended endocrine benefit from 10 vs 5 y Tamoxifen in the overall HR+ cohort (n=2,445). **Table 1**: BCI is prognostic for late distant recurrence

## Data Availability

The data analyzed in the current study are not publicly available because they contain patient data and proprietary information. Aggregated data analyzed in the study are included in the article. Qualified researchers may contact the corresponding author with reasonable requests to view additional data.

## Abbreviations

AR: Androgen receptor
AR/ER: Androgen receptor/estrogen receptor ratio
ASCO: American Society of Clinical Oncology
ATAC: Arimidex, tamoxifen, alone or in combination clinical trial
aTTom: Adjuvant tamoxifen—to offer more? Clinical trial
BCI (*H*/*I*): Breast Cancer Index (*HOXB13/IL17BR*)
BCI: Breast Cancer Index
BIG 1-98: Breast International Group 1-98 clinical trial
CAG: Confidentiality Advisory Group
CAP: College of American Pathologists
cDNA: Complementary deoxyribose nucleic acid
CLIA: Clinical Laboratory Improvement Amendments
CRCTU: University of Birmingham Cancer Research UK Clinical Trial Unit
DFI: Disease-free interval
DFS: Disease-free survival
ECRC: Edinburgh Cancer Research Center
EET: Extended endocrine therapy
ER: Estrogen receptor
FFPE: Formalin-fixed paraffin-embedded
HER2: Human epidermal growth factor receptor 2
HR: Hormone receptor
HRA: Health Research Authority
IHC: Immunohistochemical
MGI: Molecular Grade Index
N +: Node positive
N0: Node negative
PBPP: Public Benefit and Privacy Panel
PCR: Polymerase chain reaction
PR: Progesterone receptor
REC: Research Ethics Committee
RFI: Recurrence-free interval
RNA: Ribonucleic acid
RT-PCR: Reverse transcriptase polymerase chain reaction
TEAM: Tamoxifen exemestane adjuvant multinational clinical trial
TMA: Tissue microarray
Trans-aTTom: Translational aTTom study

## References

[1] Noordhoek I, Blok EJ, Meershoek-Klein Kranenbarg E, Putter H, Duijm-De Carpentier M, Rutgers EJT (2020). Overestimation of late distant recurrences in high-risk patients with ER-positive breast cancer: validity and accuracy of the CTS5 risk score in the TEAM and IDEAL trials. J Clin Oncol.

[2] Richman J, Dowsett M (2019). Beyond 5 years: enduring risk of recurrence in oestrogen receptor-positive breast cancer. Nat Rev Clin Oncol.

[3] Davies C, Pan H, Godwin J, Gray R, Arriagada R, Raina V (2013). Long-term effects of continuing adjuvant tamoxifen to 10 years versus stopping at 5 years after diagnosis of oestrogen receptor-positive breast cancer: ATLAS, a randomised trial. Lancet.

[4] Mamounas EP, Bandos H, Lembersky BC, Jeong JH, Geyer CE, Rastogi P (2019). Use of letrozole after aromatase inhibitor-based therapy in postmenopausal breast cancer (NRG Oncology/NSABP B-42): a randomised, double-blind, placebo-controlled, phase 3 trial. Lancet Oncol.

[5] Goss PE, Ingle JN, Martino S, Robert NJ, Muss HB, Piccart MJ (2005). Randomized trial of letrozole following tamoxifen as extended adjuvant therapy in receptor-positive breast cancer: updated findings from NCIC CTG MA.17. J Natl Cancer Inst.

[6] Abe O, Abe R, Enomoto K, Kikuchi K, Koyama H, Masuda H (2005). Effects of chemotherapy and hormonal therapy for early breast cancer on recurrence and 15-year survival: an overview of the randomised trials. Lancet.

[7] Pan H, Gray R, Braybrooke J, Davies C, Taylor C, McGale P (2017). 20-year risks of breast-cancer recurrence after stopping endocrine therapy at 5 years. N Engl J Med.

[8] Szostakowska M, Trębińska-Stryjewska A, Grzybowska EA, Fabisiewicz A (2019). Resistance to endocrine therapy in breast cancer: molecular mechanisms and future goals. Breast Cancer Res Treat.

[9] Allison KH, Hammond MEH, Dowsett M, McKernin SE, Carey LA, Fitzgibbons PL (2020). Estrogen and progesterone receptor testing in breast cancer: ASCO/CAP guideline update. J Clin Oncol.

[10] Huang L, Liang G, Zhang Q, Zhao W (2020). The role of long noncoding rnas in antiestrogen resistance in breast cancer: an overview and update. J Breast Cancer.

[11] Wang X, Wang S (2021). Identification of key genes involved in tamoxifen-resistant breast cancer using bioinformatics analysis. Transl Cancer Res.

[12] Dowsett M, Allred C, Knox J, Quinn E, Salter J, Wale C (2008). Relationship between quantitative estrogen and progesterone receptor expression and human epidermal growth factor receptor 2 (HER-2) status with recurrence in the arimidex, tamoxifen, alone or in combination trial. J Clin Oncol.

[13] Viale G, Regan MM, Maiorano E, Mastropasqua MG, Dell’Orto P, Rasmussen BB (2007). Prognostic and predictive value of centrally reviewed expression of estrogen and progesterone receptors in a randomized trial comparing letrozole and tamoxifen adjuvant therapy for postmenopausal early breast cancer: BIG 1-98. J Clin Oncol.

[14] Bartlett JMS, Brookes CL, Robson T, Van De Velde CJH, Billingham LJ, Campbell FM (2011). Estrogen receptor and progesterone receptor as predictive biomarkers of response to endocrine therapy: a prospectively powered pathology study in the tamoxifen and exemestane adjuvant multinational trial. J Clin Oncol.

[15] Hon JDC, Singh B, Sahin A, Du G, Wang J, Wang VY (2016). Breast cancer molecular subtypes: from TNBC to QNBC. Am J Cancer Res.

[16] Bronte G, Rocca A, Ravaioli S, Puccetti M, Tumedei MM, Scarpi E (2018). Androgen receptor in advanced breast cancer: is it useful to predict the efficacy of anti-estrogen therapy?. BMC Cancer.

[17] Elebro K, Borgquist S, Simonsson M, Markkula A, Jirström K, Ingvar C (2015). Combined androgen and estrogen receptor status in breast cancer: treatment prediction and prognosis in a population-based prospective cohort. Clin Cancer Res.

[18] Hilborn E, Gacic J, Fornander T, Nordenskjöld B, Stål O, Jansson A (2016). Androgen receptor expression predicts beneficial tamoxifen response in oestrogen receptor-α-negative breast cancer. Br J Cancer.

[19] Cochrane DR, Bernales S, Jacobsen BM, Cittelly DM, Howe EN, D’Amato NC (2014). Role of the androgen receptor in breast cancer and preclinical analysis of enzalutamide. Breast Cancer Res.

[20] Cao L, Xiang G, Liu F, Xu C, Liu J, Meng Q (2019). A high AR:ERα or PDEF:ERα ratio predicts a sub-optimal response to tamoxifen therapy in ERα-positive breast cancer. Cancer Chemother Pharmacol.

[21] Kamranzadeh H, Ardekani RM, Kasaeian A, Sadighi S, Maghsudi S, Jahanzad I, et al. Association between Ki-67 expression and clinicopathological features in prognosis of breast cancer: a retrospective cohort study. J Res Med Sci. 2019;24(1). 10.4103/jrms.JRMS_553_18 PMC6521610 31143231

[22] Liang Q, Ma D, Gao RF, Da YuK (2020). Effect of Ki-67 expression levels and histological grade on breast cancer early relapse in patients with different immunohistochemical-based subtypes. Sci Rep.

[23] Zhang Y, Schnabel CA, Schroeder BE, Jerevall PLL, Jankowitz RC, Fornander T (2013). Breast cancer index identifies early-stage estrogen receptor-positive breast cancer patients at risk for early- and late-distant recurrence. Clin Cancer Res.

[24] Bartlett JMS, Sgroi DC, Treuner K, Zhang Y, Ahmed I, Piper T (2019). Breast Cancer Index and prediction of benefit from extended endocrine therapy in breast cancer patients treated in the adjuvant tamoxifen—to offer more? (aTTom) trial. Ann Oncol.

[25] Noordhoek I, Treuner K, Putter H, Zhang Y, Wong J, Kranenbarg EMK (2021). Breast cancer index predicts extended endocrine benefit to individualize selection of patients with HRþ early-stage breast cancer for 10 years of endocrine therapy. Clin Cancer Res.

[26] Sgroi DC, Carney E, Zarrella E, Steffel L, Binns SN, Finkelstein DM (2013). Prediction of late disease recurrence and extended adjuvant letrozole benefit by the HOXB13/IL17BR biomarker. J Natl Cancer Inst.

[27] Gray RG, Rea D, Handley K, Bowden SJ, Perry P, Earl HM (2013). aTTom: long-term effects of continuing adjuvant tamoxifen to 10 years versus stopping at 5 years in 6953 women with early breast cancer. J Clin Oncol.

[28] Harvey JM, Clark GM, Osborne CK, Allred DC (1999). Estrogen receptor status by immunohistochemistry is superior to the ligand-binding assay for predicting response to adjuvant endocrine therapy in breast cancer. J Clin Oncol.

[29] Nielsen TO, Leung SCY, Rimm DL, Dodson A, Acs B, Badve S (2021). Assessment of Ki67 in breast cancer: updated recommendations from the international Ki67 in Breast Cancer Working Group. J Natl Cancer Inst.

[30] Ma XJJ, Hilsenbeck SG, Wang W, Ding L, Sgroi DC, Bender RA (2006). The HOXB13:IL17BR expression index is a prognostic factor in early-stage breast cancer. J Clin Oncol.

[31] Bartlett JMS, Sgroi DC, Treuner K, Zhang Y, Piper T, Salunga RC, et al. Breast Cancer Index is a predictive biomarker of treatment benefit and outcome from extended tamoxifen therapy: final analysis of the Trans-aTTom study. Clin Cancer Res. 2022;3385 10.1158/1078-0432.CCR-21-3385 PMC9306281 35144966

[32] Treuner K, Hayes M, Benner C, Schnabel C, Heinz S. Abstract P6-04-17: role of HOXB13 in modulating estrogen signaling in breast cancer cells. In: Cancer Research. American Association for Cancer Research (AACR); 2020. p. P6-04-17.

[33] Kim C, Tang G, Pogue-Geile KL, Costantino JP, Baehner FL, Baker J (2011). Estrogen receptor (ESR1) mRNA expression and benefit from tamoxifen in the treatment and prevention of estrogen receptor-positive breast cancer. J Clin Oncol.

[34] Mamounas EP, Bandos H, Rastogi P, Zhang Y, Treuner K, Lucas PC (2021). Breast Cancer Index (BCI) and prediction of benefit from extended aromatase inhibitor (AI) therapy (tx) in HR+ breast cancer: NRG oncology/NSABP B-42. J Clin Oncol.

[35] Fu X, Pereira R, de Angelis C, Veeraraghavan J, Nanda S, Qin L (2019). FOXA1 upregulation promotes enhancer and transcriptional reprogramming in endocrine-resistant breast cancer. Proc Natl Acad Sci U S A.

[36] Theodorou V, Stark R, Menon S, Carroll JS (2013). GATA3 acts upstream of FOXA1 in mediating ESR1 binding by shaping enhancer accessibility. Genome Res.

[37] Fortelny N, Overall CM, Pavlidis P, Freue GVC (2017). Can we predict protein from mRNA levels?. Nature.

[38] Lundin KB, Henningson M, Hietala M, Ingvar C, Rose C, Jernström H (2011). Androgen receptor genotypes predict response to endocrine treatment in breast cancer patients. Br J Cancer.

[39] Masuda H, Masuda N, Kodama Y, Ogawa M, Karita M, Yamamura J (2011). Predictive factors for the effectiveness of neoadjuvant chemotherapy and prognosis in triple-negative breast cancer patients. Cancer Chemother Pharmacol.

[40] Rangel N, Rondon-Lagos M, Annaratone L, Felipe Aristizábal-Pachon A, Cassoni P, Sapino A (2020). AR/ER ratio correlates with expression of proliferation markers and with distinct subset of breast tumors. Cells.

[41] Harbeck N, Rastogi P, Martin M, Tolaney SM, Shao ZM, Fasching PA (2021). Adjuvant abemaciclib combined with endocrine therapy for high-risk early breast cancer: updated efficacy and Ki-67 analysis from the monarchE study. Ann Oncol.

[42] Camp RL, Charette LA, Rimm DL (2000). Validation of tissue microarray technology in breast carcinoma. Lab Invest.

[43] Krauss K, Stickeler E (2020). Endocrine therapy in early breast cancer. Breast Care (Basel).

[44] Bago-Horvath Z, Rudas M, Dubsky P, Jakesz R, Singer CF, Kemmerling R (2011). Adjuvant sequencing of tamoxifen and anastrozole is superior to tamoxifen alone in postmenopausal women with low proliferating breast cancer. Clin Cancer Res.

[45] Viale G, Giobbie-Hurder A, Regan MM, Coates AS, Mastropasqua MG, Dell’Orto P (2008). Prognostic and predictive value of centrally reviewed Ki-67 labeling index in postmenopausal women with endocrine-responsive breast cancer: results from breast international group trial 1–98 comparing adjuvant tamoxifen with letrozole. J Clin Oncol.

[46] Regan MM, Pagani O, Francis PA, Fleming GF, Walley BA, Kammler R (2015). Predictive value and clinical utility of centrally assessed ER, PgR, and Ki-67 to select adjuvant endocrine therapy for premenopausal women with hormone receptor-positive, HER2-negative early breast cancer: TEXT and SOFT trials. Breast Cancer Res Treat.

[47] Beelen K, Opdam M, Severson T, Koornstra R, Vincent A, Wesseling J (2018). Mitotic count can predict tamoxifen benefit in postmenopausal breast cancer patients while Ki67 score cannot. BMC Cancer.

[48] Watanabe T, Oba T, Tanimoto K, Shibata T, Kamijo S, Ito KI (2021). Tamoxifen resistance alters sensitivity to 5-fluorouracil in a subset of estrogen receptor-positive breast cancer. PLoS ONE.

[49] NCCN Clinical Practice Guidelines in Oncology. Breast cancer, version 2.2022. National Comprehensive Cancer Network. 2022.

[50] Andre F, Ismaila N, Allison KH, Barlow WE, Collyar DE, Damodaran S (2022). Biomarkers for adjuvant endocrine and chemotherapy in early-stage breast cancer: ASCO guideline update. J Clin Oncol.

