# Supplementary material for: Correlative studies of the Breast Cancer Index (HOXB13/IL17BR) and ER, PR, AR, AR/ER ratio and Ki67 for prediction of extended endocrine therapy benefit: a Trans-aTTom study
Source: Breast Cancer Res. 2022 Dec 16;24:90. doi: 10.1186/s13058-022-01589-x (PMC9758861; doi:10.1186/s13058-022-01589-x)
Supplement: Supplementary file 1 — Additional file 1. Figure 1: DFI Benefit From 10 vs 5 y Tamoxifen is associated with increasing BCI (H/I) Levels. Figure 2: DFS benefit from 10 vs 5 y Tamoxifen is associated with increasing BCI (H/I) levels. Figure 3: Extended endocrine benefit from 10 vs 5 y Tamoxifen is not associated with Allred scores of ER, PR, or AR , or expression of ER or PR mRNA. Figure 4. Extended endocrine benefit from 10 vs 5 y Tamoxifen in the overall HR+ cohort (n=2,445). Table 1: BCI is prognostic for late distant recurrence [file 13058_2022_1589_MOESM1_ESM.docx]

**Supplemental Figure 1: DFI Benefit From 10 vs 5 y Tamoxifen is Associated with Increasing BCI (H/I) Levels**

Continuous risk curves based on DFI after 5- and 10-years of tamoxifen as a function of estrogen receptor (ER), progesterone receptor (PR), androgen receptor (AR), and Ki67 immunohistochemistry (IHC) expression, AR/ER IHC ratio and BCI (H/I) values.

**Supplemental Figure 2: DFS Benefit From 10 vs 5 y Tamoxifen is Associated with Increasing BCI (H/I) Levels**

Continuous risk curves based on DFS after 5- and 10-years of tamoxifen as a function of estrogen receptor (ER), progesterone receptor (PR), androgen receptor (AR), and Ki67 immunohistochemistry (IHC) expression, AR/ER IHC ratio and BCI (H/I) values.

**Supplemental Figure 3: Extended Endocrine Benefit From 10 vs 5 y Tamoxifen is Not Associated with Allred scores of ER, PR, or AR , or expression of ER or PR mRNA**

Continuous risk curves 5- and 10-years of tamoxifen as a function of the combined proportion and intensity of estrogen receptor (ER), progesterone receptor (PR), androgen receptor (AR), expression, and of ER and PR mRNA.

**Supplemental Figure 4. Extended Endocrine Benefit From 10 vs 5 y Tamoxifen in the overall HR+ cohort (n=2,445)**

Continuous risk curves based on RFI after 5- and 10-years of tamoxifen as a function of estrogen receptor (ER), progesterone receptor (PR), androgen receptor (AR) and Ki67 immunohistochemistry (IHC) expression, AR/ER ratio and BCI (H/I) values

**Supplemental Table 1: BCI is Prognostic for Late Distant Recurrence**

Prognostic performance of BCI; ER, PR, AR, and Ki67 protein levels; the AR/ER ratio; and ER and PR mRNA.

| Biomarker | Inter-quartile HR | P value |
| --- | --- | --- |
| BCI | 1.37 | <0.001 |
| ER IHC | 0.92 | 0.165 |
| PR IHC | 0.89 | 0.281 |
| AR IHC | 0.87 | 0.120 |
| Ki67 IHC | 0.96 | 0.489 |
| AR/ER Ratio | 0.99 | 0.484 |
| ER PCR | 1.05 | 0.441 |
| PR PCR | 1.02 | 0.811 |
